# Risk of sepsis among patients with COPD treated with fixed combinations of inhaled corticosteroids and long-acting Beta2 agonists

**DOI:** 10.18632/aging.102217

**Published:** 2019-09-10

**Authors:** Cheng-Yi Wang, You Shuei Lin, Ya-Hui Wang, Chih-Cheng Lai, Hao-Chien Wang, Likwang Chen, Chong-Jen Yu, on behalf of Taiwan Clinical Trial Consortium for Respiratory Diseases (TCORE)

**Affiliations:** 1 Department of Internal Medicine, Cardinal Tien Hospital and School of Medicine, College of Medicine, Fu Jen Catholic University, New Taipei City, Taiwan; 2 Department of Physiology, School of Medicine, College of Medicine, Taipei Medical University, Taipei, Taiwan; 3 Medical Research Center, Cardinal Tien Hospital and School of Medicine, College of Medicine, Fu Jen Catholic University, New Taipei City, Taiwan; 4 Department of Internal Medicine, Kaohsiung Veterans General Hospital, Tainan Branch, Tainan, Taiwan; 5 Department of Internal Medicine, National Taiwan University Hospital and College of Medicine, National Taiwan University, Taipei, Taiwan; 6 Institute of Population Health Sciences, National Health Research Institutes, Zhunan, Miaoli County, Taiwan

**Keywords:** sepsis, chronic obstructive pulmonary disease, inhaled corticosteroids, long-acting beta2 agonists

## Abstract

This study aimed to compare the effect of budesonide/formoterol and fluticasone/salmeterol on the risk and outcomes of sepsis in COPD patients. We conducted this study using the Taiwan National Health Insurance Research Database. We included COPD patients prescribed with budesonide/formoterol or fluticasone/salmeterol between 2004 and 2011. Outcomes including sepsis and mortality were measured. 10,267 COPD patients who received fluticasone/salmeterol and 6,844 patients who received budesonide/formoterol were enrolled into this study and then subsequence were adjusted by propensity score weighting. The incidence of sepsis was 5.74 and 4.99 per 100 person-years for the patients receiving fluticasone/salmeterol and budesonide/formoterol, respectively. Fluticasone/salmeterol was associated with higher risk of sepsis (aHR, 1.15; 95%CI, 1.07-1.24) and septic shock (aHR, 1.14; 95%CI, 1.01-1.29) than budesonide/formoterol. Besides, fluticasone/salmeterol was associated with higher risk of death (aHR, 1.090; 95%CI, 1.01-1.18) than budesonide/formoterol. Patients receiving fluticasone/salmeterol had a significant higher risk of sepsis related respiratory organ dysfunction, lower respiratory tract infection, genitourinary tract infection, bacteremia and skin infection. In conclusion, long-term treatment with budesonide/formoterol was associated with lower rates of sepsis and deaths than fluticasone/salmeterol in patients with COPD.

## INTRODUCTION

COPD is a common cause of death in the whole world [1], and the incidence of COPD continue to increase [2]. Respiratory tract infections are a common trigger of COPD exacerbations, and are associated with increased rates of morbidity and mortality. For COPD patients, inhaled corticosteroid (ICS) treatment is important maintenance therapy, and the combination of inhaled long-acting β2-agonists (LABAs) and ICSs can effectively reduce the risk of exacerbations among COPD patients [3–7]. In addition to the clinical benefits of ICSs, the use of ICSs can increase the risk of pneumonia – a common precursor of sepsis in patients with COPD [8–12]. Moreover, COPD patients with sepsis had a higher risk of severe exacerbations, pneumonia, and death compared to those without sepsis [22]. Despite most previous studies have focused on the association between ICSs and pneumonia or serious pneumonia, only a few have investigated the association between ICS treatment and the risk of sepsis [13]. In contrast to the significant association between ICS and the risk of pneumonia, one large-cohort study [13] using the administrative health databases showed that the risk of sepsis is not increased with ICS among patients treated for COPD. Therefore, the association between ICS used and sepsis risk remains unclear, and further study is warranted to clarify this issue.

Several studies [7, 14, 15] have evaluated differences between budesonide/formoterol and fluticasone/salmeterol. For example, Blais et al. found that budesonide/formoterol was associated with less COPD related emergency department visits and hospitalizations and fewer uses of anticholinergic medications than fluticasone/salmeterol [15]. In addition, Janson et al. reported higher rates of pneumonia and pneumonia-related mortality in fluticasone/salmeterol users than budesonide/formoterol users [7]. Further, Larsson et al. demonstrated that budesonide/formoterol was better than fluticasone/salmeterol in reducing the risk of exacerbations in COPD patients [14]. However, the relative risk of severe sepsis between patients with COPD receiving these two combinations is unknown. Therefore, we compared the effects of budesonide/formoterol and fluticasone/salmeterol on the sepsis risk in propensity score-matched COPD patients using data based on Taiwan National Health Insurance Research Database (NHIRD).

## RESULTS

### Characteristics of the study population

**Figure 1:**
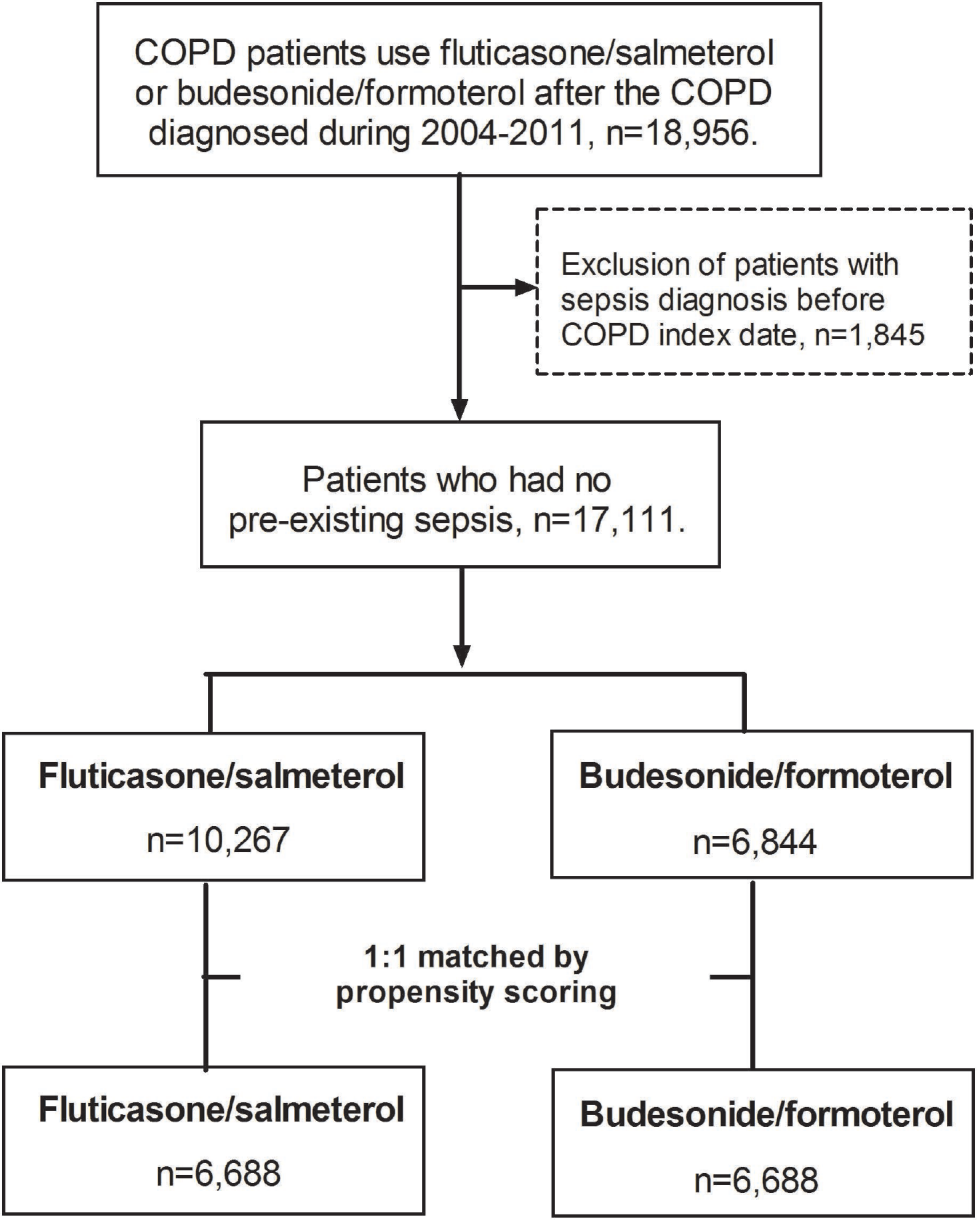
**Flowchart of study cohort selection.**

Initially, a total of 17,111 patients had the diagnosis of COPD and received a fixed ICS/LABA combination (10,267 patients with fluticasone/salmeterol and 6,844 patients with budesonide/formoterol) were identified after excluding those with previous sepsis. Then, pairwise matching (1:1) resulted in two similar subgroups, with 6,688 patients in each (Figure 1). After propensity score matching, no significant difference was found between the patients who received fluticasone/salmeterol and those who received budesonide/formoterol for all covariates.

### Risk of sepsis and death

**Figure 2:**
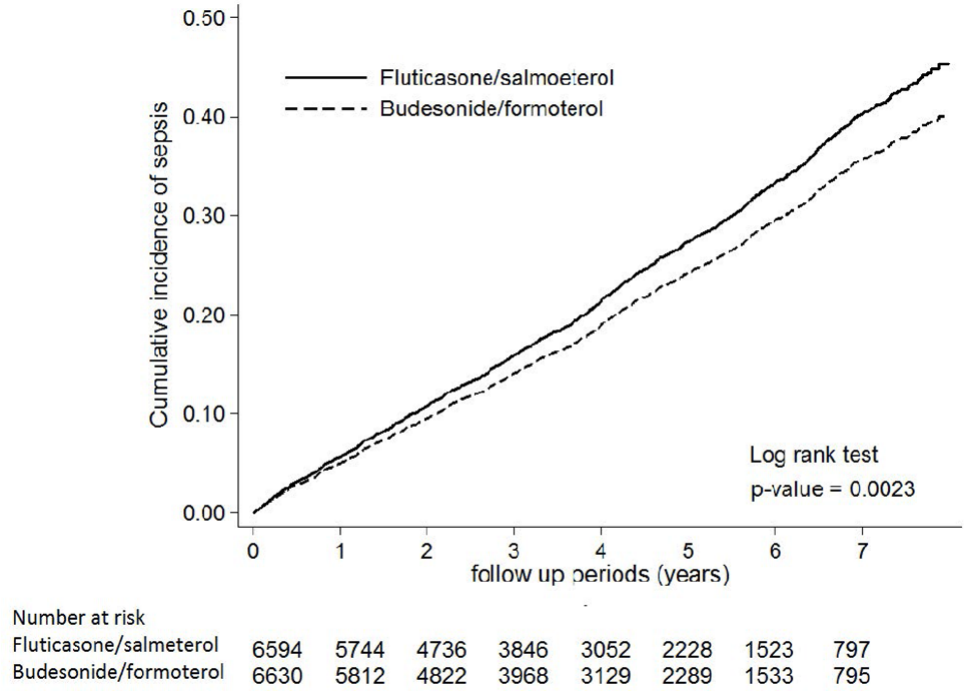
**Cumulative incidence curve for sepsis in the patients prescribed with fluticasone/salmeterol and budesonide/formoterol.** Times to events were compared using log-rank tests.

Table 1 showed event rates of sepsis and mortality of COPD patients prescribed with fluticasone/salmeterol and budesonide/formoterol. During the follow-up period, the incidence of sepsis was 5.74 per 100 person-years for fluticasone/salmeterol group and 4.99 per 100 person-years for budesonide/formoterol group. Fluticasone/salmeterol was associated with a higher risk of sepsis (aHR, 1.15; 95% CI, 1.07-1.24), septic shock (aHR, 1.14; 95% CI, 1.01-1.29) and mortality (aHR, 1.09; 95% CI, 1.01-1.18), than budesonide/formoterol. Furthermore, the cumulative incidence of sepsis was higher among the patients receiving fluticasone/salmeterol than in those receiving budesonide/formoterol (p = 0.0001) (Figure 2). All these differences remained significant in the competing risk analysis (Table 2). Among as-treated analysis, fluticasone/salmeterol was associated with a higher risk of sepsis and death compared to budesonide/formoterol. In the competing risk analysis, the risk of sepsis was similar between the two groups, the patients receiving fluticasone/salmeterol had a trend of higher risk of sepsis than those receiving budesonide/formoterol.

**Table 1:** Incidences (per 100 person-years) and risk of sepsis and mortality among fluticasone/salmeterol and budesonide/formoterol.

	**Fluticasone/salmeterol cohort**			**Budesonide/formoterol cohort**			**Crude HR (95%CI)**	**Adjusted^b^ HR (95%CI)**	**Competing risk HR (95%CI)**
	**event**	**Person-year**	**IR^a^**	**event**	**Person-year**	**IR^a^**			
Mortality	1242	27636.96	4.49	1151	27801.28	4.14	1.09 (1.00-1.18)	1.09 (1.01-1.18)	-
Sepsis	1449	25229.12	5.74	1281	25685.67	4.99	1.15 (1.07-1.24)	1.15 (1.07-1.24)	1.13 (1.05-1.22)
Septic shock	543	23103.66	2.35	488	23743.05	2.06	1.14 (1.01-1.29)	1.14 (1.01-1.29)	1.14 (1.01-1.28)

^a^IR, incidence rate, per 100 person-years.

^b^adjusted for propensity score.

**Table 2:** Sensitivity analyses for risk of sepsis and mortality among fluticasone/salmeterol and budesonide/formoterol.

**Sensitivity analyses**	**Crude HR (95%CI)**	**Adjusted^a^ HR (95%CI)**
Primary analysis		
Mortality	1.086(1.002-1.176)	1.090(1.006-1.181)
Sepsis	1.150(1.067-1.240)	1.153(1.069-1.243)
Septic shock	1.143(1.012-1.292)	1.143(1.012-1.292)
ITT analysis + competing risk		
Mortality	-	-
Sepsis		1.131(1.047-1.222)
Septic shock		1.136(1.005-1.284)
As-treated analysis		
Mortality	1.237(1.095-1.398)	1.227(1.086-1.387)
Sepsis	1.177(1.048-1.321)	1.173(1.045-1.317)
Septic shock	1.208(1.01-1.445)	1.201(1.004-1.437)
As-treated analysis + competing risk		
Mortality	-	-
Sepsis		1.118(0.988-1.266)
Septic shock		1.188(0.993-1.422)

### Risk of sepsis-related organ dysfunction and infection types

Fluticasone/salmeterol was associated with a higher risk of sepsis related respiratory organ dysfunction than budesonide/formoterol. Although the other organ dysfunction had the same trends, the difference did not reach statistical significance. Moreover, fluticasone/salmeterol was associated with higher risk of several types of infections, including lower respiratory tract infection, genitourinary tract infection, bacteremia and skin infection than budesonide/formoterol (Table 3).

**Table 3:** Incidences (per 100 person-years) of severe sepsis-related organ dysfunction and infection types in a COPD patients prescribed with fluticasone/salmeterol and budesonide/formoterol after propensity score matching.

**Variables**	**Fluticasone/salmeterol cohort**			**Budesonide/formoterol cohort**			**Crude HR (95%CI)**	Adjusted* HR (95%CI)	**Competing risk HR (95%CI)**
	**event**	**Person-year**	**IR^a^**	**event**	**Person-year**	**IR^a^**			
Number of organ dysfunction									
1	1232	24663.40	5.00	1100	25219.33	4.36	1.143 (1.054-1.24)	1.145 (1.055-1.242)	1.138 (1.049-1.235)
2+	217	22425.39	0.97	181	23061.08	0.78	1.233 (1.012-1.501)	1.239 (1.017-1.51)	1.23 (1.009-1.498)
Site of organ dysfunction									
Respiratory	1167	24586.69	4.75	989	24923.85	3.97	1.195 (1.098-1.300)	1.195 (1.098-1.301)	1.189 (1.093-1.295)
Cardiovascular	178	22295.01	0.80	185	23099.32	0.80	0.996 (0.811-1.223)	0.999 (0.813-1.227)	0.991 (0.807-1.218)
Renal	158	22251.54	0.71	139	22979.47	0.60	1.173 (0.934-1.473)	1.178 (0.938-1.480)	1.169 (0.930-1.468)
Hepatic	57	21992.90	0.26	57	22701.64	0.25	1.034 (0.716-1.492)	1.035 (0.717-1.493)	1.029 (0.713-1.485)
Neurologic	68	22017.01	0.31	64	22769.88	0.28	1.100 (0.782-1.547)	1.103 (0.784-1.552)	1.094 (0.778-1.540)
Hematologic	30	21939.09	0.14	22	22642.32	0.10	1.408 (0.812-2.441)	1.411 (0.814-2.446)	1.400 (0.808-2.428)
Metabolic	36	21940.47	0.16	36	22694.06	0.16	1.036 (0.653-1.645)	1.037 (0.653-1.646)	1.030 (0.649-1.634)
Infection									
Lower respiratory tract infection	1301	24872.75	5.23	1166	25392.32	4.59	1.138 (1.051-1.231)	1.140 (1.053-1.233)	1.133 (1.047-1.226)
Genitourinary tract infection	215	22358.56	0.96	159	23007.37	0.69	1.391 (1.133-1.707)	1.398 (1.139-1.716)	1.387 (1.130-1.702)
Intra-abdominal infection	25	21928.84	0.11	24	22651.00	0.11	1.074 (0.613-1.880)	1.075 (0.614-1.882)	1.068 (0.609-1.871)
Dermatologic infection	14	21890.51	0.06	5	22602.40	0.02	2.885 (1.039-8.010)	2.884 (1.039-8.008)	2.871 (1.035-7.964)
Other bacterial diseases	54	21982.45	0.25	50	22691.87	0.22	1.115 (0.759-1.639)	1.117 (0.760-1.641)	1.111 (0.756-1.631)
Invasive fungal infections	49	21961.38	0.22	51	22691.22	0.22	0.992 (0.670-1.469)	0.993 (0.671-1.470)	0.988 (0.667-1.462)
Bacteremia	258	22491.13	1.15	220	23161.93	0.95	1.206 (1.008-1.444)	1.210 (1.011-1.449)	1.202 (1.004-1.439)
Tuberculosis	33	21939.67	0.15%	38	22670.49	0.17	0.899 (0.564-1.433)	0.900 (0.565-1.435)	0.894 (0.561-1.425)
Biliary tract infection	10	21876.33	0.05	8	22612.07	0.04	1.293 (0.510-3.275)	1.292 (0.510-3.275)	1.287 (0.508-3.259)

^a^IR indicates incidence rate, per 100 person-year.

^·^ Adjusted for propensity score.

## DISCUSSION

In this study, the COPD patients receiving fluticasone/salmeterol had a higher risk of sepsis, septic shock and death than those receiving budesonide/formoterol. Similar trends were obtained using intention-to-treat and as-treated analyses with or without competing risk analysis. Moreover, using fluticasone/salmeterol was associated with higher risk of sepsis-related organ dysfunction, and most types of infections than budesonide/formoterol. A cohort study in Italy [16] reported that the incidence of hospitalization, oral corticosteroid use, and antibiotic use was lower for budesonide/formoterol compared to fluticasone/salmeterol. Another retrospective claims-based study from major U.S. health plans [17] reported that COPD patients receiving budesonide/formoterol treatment had lower average COPD-related, pneumonia-related, and all-cause costs compared to those receiving fluticasone/salmeterol treatment. In addition, the PATHOS study reported that budesonide/formoterol was associated with the lower rate of COPD-related hospitalization, and shorter hospital stay for exacerbations compared with fluticasone/salmeterol. In contrast, fluticasone/salmeterol was associated with higher risk of pneumonia and more pneumonia-related hospital admissions compared to the budesonide/formoterol group [7, 14]. Taken together, these findings may suggest that budesonide/formoterol treatment is associated with fewer adverse effects compared with fluticasone/salmeterol treatment. By this study, our findings add to the growing evidence of significant differences between fluticasone/salmeterol and budesonide/formoterol in terms of adverse effects in patients with COPD.

The following differences between budesonide/formoterol and fluticasone/salmeterol can support our findings. Budesonide and fluticasone differ in their pharmacokinetic properties, and the rates of uptake and elimination are slower for fluticasone [18]. In addition, the mean residence time in the systemic circulation has been reported to be shorter for budesonide than for fluticasone (4.41 hours versus 12.78 hours, respectively), and the quantity of expectorated fluticasone has been reported to be significantly higher compared to budesonide (ratio 5.21; P = 0.006) [20]. Fluticasone has been reported to be approximately 10 times more potent than budesonide in inhibiting the release of IL-6, IL-8, and TNF-α production from human alveolar macrophages [20]. In summary, all these differences can make fluticasone retention longer in the airway and exhibit greater immunosuppressive effect than budesonide, and thereby fluticasone can be associated with higher risk of bacterial colonization and infection-associated exacerbations than budensonide [18–20]. Other than the pharmacokinetic differences between budesonide and fluticasone, their differences in individual dosage might be related the risk of sepsis. Based on the findings of the study [22] investigating the association between ICS and risk of serious pneumonia, the risk was twofold with fluticasone (RR 2.01; 95% CI 1.93 to 2.10), and the risk was increasing with the daily dose. In contrast, the risk of serious pneumonia was only 1.17 with budesonide (95% CI 1.09 to 1.26), and the dose-response relationship was not as evident as fluticasone. Moreover, this study [22] also demonstrated that the effect of ICS on the risk of pneumonia would peak in the first year of use and remains elevated and stable even up to 5 years of continuous use.

This study has the major strength. A large COPD cohort in a real world was enrolled in this study and the effect of many confounding factor were minimized. Thus, our results could reveal the status of COPD patients in Taiwan and could be generalized to other sites.

This study has several limitations. First, the result of pulmonary function test results was not available, so the severity of COPD was not determined. Second, although many confounding factors was collected and adjusted in this study, it is still possible that residual confounding factors were exists.

In summary, long-term treatment with budesonide/formoterol was associated with lower rates of sepsis, septic shock and death than fluticasone/salmeterol in COPD patients. Clinicians should keep alert higher risk of sepsis and death among COPD patients receiving fluticasone/salmeterol than budesonide/formoterol.

## METHODS

### Patients

A subset of the NHIRD including data on 2,200,000 COPD patient was used for this study. This population was followed between 2004 and 2011. International Classification of Diseases, Ninth Revision, Clinical Modification (ICD-9-CM) codes 491, 492, and 496 were used to identify patients with COPD. Only patients with at least three outpatient or inpatient visits for COPD and those aged between 40 and 100 years were included. We defined the index date as the date of the first fixed prescription for a combination of ICSs/LABAs for COPD. The patients were followed from 1 January 2004 to the either one of the following outcomes, including the end of the study (31 December 2011), emigration or mortality. Ethical approval was obtained from the Institutional Review Board (No. CTH-104-3-5-030).

### Outcome measurements

The primary outcome was sepsis, which was defined using ICD-9-CM codes as previously reported.^16^ Only the first episode of sepsis was included but those with previous sepsis were excluded. The secondary outcome was all-cause mortality. Moreover, the source of infection, and organ dysfunction were recorded as previous reported, [23] Sepsis patients received sympathomimetic or vasopressin were defined as septic shock.

### Exposure measures and potential confounders

Comorbidity data were retrieved according to ICD-9-CM codes and the severity of the comorbidities was assessed using Charlson Comorbidity Index (CCI). Concomitant medications, particularly medications for COPD (LABAs, short-acting β2-agonists (SABAs), long-acting muscarinic antagonists (LAMAs), and ICSs), were recorded. Fixed ICS/LABA combinations were defined as ATC codes R03AK06 or R03AK07. COPD exacerbations were only recorded during the period when the ICS/LABA combinations were prescribed.

### Statistical analysis

Propensity score method was generated for the likelihood of fluticasone/salmeterol use (with budesonide/formoterol use as reference group). A logistic regression with covariates of index year, age, sex, monthly income, hospital level, the number of outpatient visits, the use of concomitant medication, and underlying comorbidities (Supplementary Table 1) was used to compute the propensity scores. We used cox regression models to assess the crude and adjusted hazard ratios (HRs) of the outcomes in the two groups for propensity scores (continuous). The budesonide/formoterol group was used as the reference group. The crude incidence of sepsis was defined as the total episodes of events divided by the person-years.

This study used intention-to-treat analysis as the primary analysis. In the as-treated analysis, the patients were censored when they switched to another ICS/LABA and when they out of the study. We used Kaplan-Meier method to construct cumulative incidence curves.

We used SAS software version 9.4 (SAS Institute Inc., Cary, NC) for data analysis. A two-sided P value < 0.05 indicated statistical significance in all analyses.

## Supplementary Material

Supplementary Table
