# Supplementary material for: Risk of sepsis among patients with COPD treated with fixed combinations of inhaled corticosteroids and long-acting Beta2 agonists
Source: Aging (Albany NY). 2019 Sep 10;11(17):6863–71. doi: 10.18632/aging.102217 (PMC6756880; doi:10.18632/aging.102217)
Supplement: Supplementary Table [file aging-11-102217-s001.docx]

SUPPLEMENTARY Table

**Supplementary Table 1. Baseline characteristics of the study population with a first ICS/LABA prescription after a diagnosis of COPD**

| Variables | Before matching | | | |  | After matching | | | |  |
| --- | --- | --- | --- | --- | --- | --- | --- | --- | --- | --- |
|  | Fluticasone/salmeterol | | Budesonide/formoterol | | *p*-value | Fluticasone/salmeterol | | Budesonide/formoterol | | *p*-value |
|  | cohort | | cohort | |  | cohort | | cohort | |  |
| **No. of patients** | 10,267 | | 6,844 | |  | 6,688 | | 6,688 | |  |
| **Index year, n (%)** |  |  |  |  | <.0001 |  |  |  |  | 0.9939 |
| 2004 | 1792 | 17.45% | 1325 | 19.36% |  | 1301 | 19.45% | 1299 | 19.42% |  |
| 2005 | 1144 | 11.14% | 1117 | 16.32% |  | 1023 | 15.30% | 1039 | 15.54% |  |
| 2006 | 1192 | 11.61% | 926 | 13.53% |  | 912 | 13.64% | 902 | 13.49% |  |
| 2007 | 1337 | 13.02% | 896 | 13.09% |  | 903 | 13.50% | 884 | 13.22% |  |
| 2008 | 1221 | 11.89% | 773 | 11.29% |  | 734 | 10.97% | 761 | 11.38% |  |
| 2009 | 1352 | 13.17% | 711 | 10.39% |  | 703 | 10.51% | 709 | 10.60% |  |
| 2010 | 1380 | 13.44% | 746 | 10.90% |  | 754 | 11.27% | 744 | 11.12% |  |
| 2011 | 849 | 8.27% | 350 | 5.11% |  | 358 | 5.35% | 350 | 5.23% |  |
| **Age (years), mean ± SD** | 65.74±10.27 | | 63.04±10.41 | | <.0001 | 63.44±1.52 | | 63.40±1.53 | | 0.7895 |
| **Male gender, n (%)** | 7844 | 76.40% | 4985 | 72.84% | <.0001 | 4913 | 73.46% | 4893 | 73.16% | 0.6958 |
| **Monthly income (NTD), n (%)** |  |  |  |  | 0.0019 |  |  |  |  | 0.764 |
| <19,100 | 3585 | 34.92% | 2259 | 33.01% |  | 2205 | 32.97% | 2232 | 33.37% |  |
| 19,100-41,999 | 5344 | 52.05% | 3581 | 52.32% |  | 3495 | 52.26% | 3495 | 52.26% |  |
| ≧42,000 | 1338 | 13.03% | 1004 | 14.67% |  | 988 | 14.77% | 961 | 14.37% |  |
| **Hospital level, n (%)** |  |  |  |  | <.0001 |  |  |  |  | 0.9983 |
| Level 1 | 4146 | 40.38% | 2846 | 41.58% |  | 2781 | 41.58% | 2779 | 41.55% |  |
| Level 2 | 4369 | 42.55% | 2638 | 38.54% |  | 2606 | 38.97% | 2615 | 39.10% |  |
| Level 3 | 1292 | 12.58% | 948 | 13.85% |  | 918 | 13.73% | 913 | 13.65% |  |
| Level 4 | 460 | 4.48% | 412 | 6.02% |  | 383 | 5.73% | 381 | 5.70% |  |
| **Outpatient visits, n (%)** |  |  |  |  | 0.1366 |  |  |  |  | 0.8747 |
| 0-5 visits | 4429 | 43.14% | 2885 | 42.15% |  | 2789 | 41.70% | 2813 | 42.06% |  |
| 6-10 visits | 2392 | 23.30% | 1667 | 24.36% |  | 1663 | 24.87% | 1623 | 24.27% |  |
| 11-15 visits | 2415 | 23.52% | 1652 | 24.14% |  | 1601 | 23.94% | 1619 | 24.21% |  |
| >15 visits | 1031 | 10.04% | 640 | 9.35% |  | 635 | 9.49% | 633 | 9.46% |  |
| **Severe exacerbations (count 1 year before the index date), n (%)** |  |  |  |  | <.0001 |  |  |  |  | 0.866 |
| 0 | 6092 | 59.34% | 4422 | 64.61% |  | 4308 | 64.41% | 4291 | 64.16% |  |
| 1 | 1718 | 16.73% | 1009 | 14.74% |  | 976 | 14.59% | 998 | 14.92% |  |
| 2+ | 2457 | 23.93% | 1413 | 20.65% |  | 1404 | 20.99% | 1399 | 20.92% |  |

| Variables | Before matching | | | |  | After matching | | | |  |
| --- | --- | --- | --- | --- | --- | --- | --- | --- | --- | --- |
|  | Fluticasone/salmeterol | | Budesonide/formoterol | | *p*-value | Fluticasone/salmeterol | | Budesonide/formoterol | | *p*-value |
|  | cohort | | cohort | |  | cohort | | cohort | |  |
| **Medication for hypertension** |  |  |  |  |  |  |  |  |  |  |
| Alpha-Blocker | 1086 | 10.58% | 676 | 9.88% | 0.1398 | 669 | 10.00% | 669 | 10.00% | 1 |
| Beta-Blocker | 2788 | 27.15% | 1731 | 25.29% | 0.0068 | 1707 | 25.52% | 1714 | 25.63% | 0.8897 |
| Calcium-Channel Blocker | 4856 | 47.30% | 3018 | 44.10% | <.0001 | 3002 | 44.89% | 2982 | 44.59% | 0.728 |
| Diuretic | 3622 | 35.28% | 2113 | 30.87% | <.0001 | 2102 | 31.43% | 2095 | 31.32% | 0.8962 |
| ACEI or ARB | 3606 | 35.12% | 2237 | 32.69% | 0.001 | 2233 | 33.39% | 2201 | 32.91% | 0.5567 |
| **Other Medication** |  |  |  |  |  |  |  |  |  |  |
| Aspirin | 1337 | 13.02% | 728 | 10.64% | <.0001 | 729 | 10.90% | 725 | 10.84% | 0.9115 |
| Clopidogrel | 506 | 4.93% | 288 | 4.21% | 0.0282 | 299 | 4.47% | 287 | 4.29% | 0.6122 |
| Ticlopidine | 197 | 1.92% | 101 | 1.48% | 0.03 | 97 | 1.45% | 101 | 1.51% | 0.7746 |
| Dipyridamole | 1358 | 13.23% | 796 | 11.63% | 0.002 | 820 | 12.26% | 789 | 11.80% | 0.41 |
| Nitrate | 121 | 1.18% | 65 | 0.95% | 0.1574 | 56 | 0.84% | 63 | 0.94% | 0.5192 |
| Statin | 1267 | 12.34% | 824 | 12.04% | 0.5562 | 810 | 12.11% | 816 | 12.20% | 0.8739 |
| NSAID | 7824 | 76.21% | 5277 | 77.10% | 0.174 | 5167 | 77.26% | 5155 | 77.08% | 0.8048 |
| Anti-hyperglycemic drugs | 1540 | 15.00% | 883 | 12.90% | 0.0001 | 860 | 12.86% | 880 | 13.16% | 0.6072 |
| PPI (Proton-pump inhibitor) | 1138 | 11.08% | 693 | 10.13% | 0.0469 | 673 | 10.06% | 688 | 10.29% | 0.6679 |
| **COPDDRUG** |  |  |  |  |  |  |  |  |  |  |
| Oral steroids | 6068 | 59.10% | 4292 | 62.71% | <.0001 | 4169 | 62.34% | 4168 | 62.32% | 0.9858 |
| Antibiotics | 7126 | 69.41% | 4856 | 70.95% | 0.0306 | 4715 | 70.50% | 4732 | 70.75% | 0.7469 |
| LABA | 370 | 3.60% | 330 | 4.82% | <.0001 | 309 | 4.62% | 314 | 4.69% | 0.8375 |
| SABA | 2969 | 28.92% | 2261 | 33.04% | <.0001 | 2148 | 32.12% | 2177 | 32.55% | 0.5919 |
| LAMA | 1280 | 12.47% | 629 | 9.19% | <.0001 | 631 | 9.43% | 628 | 9.39% | 0.9292 |
| Theophylline | 7161 | 69.75% | 4905 | 71.67% | 0.0069 | 4764 | 71.23% | 4779 | 71.46% | 0.7742 |
| Aminophylline | 4229 | 41.19% | 2868 | 41.91% | 0.3523 | 2787 | 41.67% | 2794 | 41.78% | 0.9023 |
| ICS | 2772 | 27.00% | 2346 | 34.28% | <.0001 | 2194 | 32.81% | 2228 | 33.31% | 0.532 |

| Variables | Before matching | | | |  | After matching | | | |  |
| --- | --- | --- | --- | --- | --- | --- | --- | --- | --- | --- |
|  | Fluticasone/salmeterol | | Budesonide/formoterol | | *p*-value | Fluticasone/salmeterol | | Budesonide/formoterol | | *p*-value |
|  | cohort | | cohort | |  | cohort | | cohort | |  |
| **Baseline Comorbidities** |  |  |  |  |  |  |  |  |  |  |
| Charlson Score | 1.60 | ±0.98 | 1.52 | ±0.94 | <.0001 | 1.52 | ±0.88 | 1.53 | ±0.88 | 0.7914 |
| Myocardial infarction | 152 | 1.48% | 78 | 1.14% | 0.0579 | 80 | 1.20% | 78 | 1.17% | 0.8728 |
| Congestive heart failure | 802 | 7.81% | 473 | 6.91% | 0.0280 | 459 | 6.86% | 468 | 7.00% | 0.7593 |
| Peripheral vascular disease | 75 | 0.73% | 45 | 0.66% | 0.5752 | 46 | 0.69% | 45 | 0.67% | 0.9162 |
| Cerebrovascular disease | 499 | 4.86% | 255 | 3.73% | 0.0004 | 260 | 3.89% | 253 | 3.78% | 0.7526 |
| Dementia | 140 | 1.36% | 59 | 0.86% | 0.0027 | 50 | 0.75% | 59 | 0.88% | 0.3867 |
| Reumatologic disease | 89 | 0.87% | 63 | 0.92% | 0.714 | 61 | 0.91% | 61 | 0.91% | 1 |
| Peptic ulcer disease | 1478 | 14.40% | 927 | 13.54% | 0.1167 | 899 | 13.44% | 915 | 13.68% | 0.6862 |
| Hemiplegia or paraplegia | 7 | 0.07% | 2 | 0.03% | 0.2762 | 4 | 0.06% | 2 | 0.03% | 0.4141 |
| Renal disease | 218 | 2.12% | 124 | 1.81% | 0.1538 | 126 | 1.88% | 121 | 1.81% | 0.7481 |
| Diabetes | 1073 | 10.45% | 630 | 9.21% | 0.0077 | 604 | 9.03% | 625 | 9.35% | 0.5296 |
| Moderate or Severe liver disease | 330 | 3.21% | 213 | 3.11% | 0.7093 | 212 | 3.17% | 207 | 3.10% | 0.804 |
| Tumor | 338 | 3.29% | 185 | 2.70% | 0.0283 | 188 | 2.81% | 182 | 2.72% | 0.7518 |
